# GnRH agonist in association with hCG versus hCG alone for final oocyte maturation triggering in GnRH antagonist cycles

**DOI:** 10.5935/1518-0557.20200089

**Published:** 2021

**Authors:** Condesmar M. de Oliveira Filho, Carlos A. M. de Oliveira, Larissa L. Fonseca, Kelly R. R. de Souza, Moacir R.M. Radaelli

**Affiliations:** 1 Núcleo Santista de Reprodução Humana, Santos - SP, Brazil; 2 Urology Department, Medical School, Faculdade Ingá, Maringá, Brazil

**Keywords:** Oocyte, dual triggering, gonadotropin-releasing hormone, GnRH agonist, GnRH antagonist, human chorionic gonadotropin

## Abstract

**Objective:**

To analyze gonadotropin-releasing hormone (GnRH) agonist in association with human chorionic gonadotropin (hCG) (dual triggering) versus hCG alone (conventional triggering) for final oocyte maturation triggering in GnRH antagonist cycles in an unselected population of Brazilian women.

**Methods:**

This prospective case-control study involved 114 patients referred to autologous *in vitro* fertilization treatment between February 2018 and August 2019, recruited regardless of age, infertility factor or number of cycles. The patients were randomly allocated into two groups according to oocyte maturation triggering approach: group A (n = 48) - hCG only; and group B (n = 66) - hCG plus GnRH agonist. The main outcomes measured were the number of total and metaphase II (MII) oocytes retrieved.

**Results:**

The groups were homogenous in terms of age. There were no moderate or severe ovarian hyperstimulation syndrome events. There were no statistical differences concerning total or MII oocytes retrieved between the groups (*p* > 0.05). The MII/total oocyte rate was 70.9% in group A, and 74.5% in group B (*p* = 0.679). There was no oocyte retrieved in 2/48 patients (4.16%) in group A, 1/66 (1.5%) in group B. There were no MII oocytes in 4/48 patients (8.3%) in group A, and 2/66 (3%) in group B. Age was directly correlated to the number of total and MII oocytes retrieved (*p* < 0.05).

**Conclusions:**

Dual triggering was equivalent to conventional hCH triggering in terms of the number of total and MII oocytes retrieved in the general population. Further studies are necessary to ascertain dual triggering indication in selected groups of women.

## INTRODUCTION

Controlled ovarian hyperstimulation (COH) is one of the most important strategies involved in successful in vitro fertilization-embryo transfers (IVF-ET) (Penzias, 2004). At the end of COH, human chorionic gonadotropin (hCG) is usually used as a surrogate LH surge to trigger the last stage of oocyte maturation, so that they can be retrieved and fertilized (Ludwig *et al*., 2003). However, some studies have suggested that hCG can have a negative impact on endometrial receptivity (Simon *et al*., 1995; 1998; Forman et al., 1998) and embryo quality (Valbuena *et al*., 2001; Tavaniotou *et al*., 2002). Moreover, hCG has also been associated with increased risks of ovarian hyperstimulation syndrome (OHSS) in gonadotropin-releasing hormone (GnRH) antagonist IVF cycles (Delvigne & Rozenberg, 2003).

In COH regimens with GnRH antagonists to down-regulate the cycle, GnRH agonists can be used alternatively to hCG to trigger the release of endogenous luteinizing hormone (LH) (Gonen *et al*., 1990; Tay, 2002). In this approach, the pituitary remains responsive to the GnRH agonist (Felberbaum *et al*., 1995; Orvieto *et al*., 2006), very similar to what happens in natural cycles (Nevo *et al*., 2003; Kol, 2004). Among the advantages of using GnRH agonists compared to hCG, we find the reduced risk of OHSS (Kol, 2004), a more physiological LH and FSH surge, which may result in improved oocyte (Humaidan *et al*., 2005) and endometrial quality (Forman *et al*., 1998; Simon *et al*., 1998). Nevertheless, a recent Cochrane analysis indicated that GnRH agonists alone should not be routinely used for final oocyte maturation, due to lower live birth rates and lower ongoing pregnancy rates (Youssef *et al*., 2014), which have been attributed to defective luteal phase function (Engmann & Benadiva, 2010).

Recently, a novel strategy to modify ovulation triggering through the use of a bolus of GnRH agonist plus a reduced or standard dosage of hCG (dual trigger) was proposed (Shapiro *et al*., 2011; Griffin *et al*., 2014; Jung *et al*., 2014; Kim *et al*., 2014; O’Neill *et al*., 2016). The rationale behind this strategy was that triggering with a GnRH agonist would effectively minimize the risk of OHSS, while the added hCG would also preserve adequate luteal function. Moreover, it has been suggested that the dual trigger approach can also result in better oocyte maturation, blastulation and pregnancy rates (Shapiro *et al*., 2011; Lin *et al*., 2013; Kim *et al*., 2014; Orvieto, 2015).

However, whether the addition of hCG on the day of GnRH agonist triggering actually produces better oocyte retrieval outcomes remains controversial. In an attempt to elucidate this question, two systematic reviews and meta-analyses were recently conducted (Ding *et al*., 2017; Chen *et al*., 2018). In both cases, although the authors indicated that dual triggering seemed to be more favorable at improving pregnancy rates, it was equivalent to the hCG triggering in terms of the number of oocytes and mature oocytes retrieved. Because mature oocytes are a prerequisite in IVF cycles, further studies are required to better elucidate the most effective protocol.

Therefore, the objective of the present study was to analyze GnRH agonist in association with hCG (dual triggering) versus hCG alone (conventional triggering) for final oocyte maturation triggering in GnRH antagonist cycles in a population of unselected Brazilian women.

## MATERIALS AND METHODS

### Study population

All patients admitted at a private fertility clinic in the city of Santos (Brazil) for autologous in vitro fertilization (IVF) treatment between February 2018 and August 2019, were recruited regardless of age, infertility factor (male factor, unexplained infertility, tubal factor infertility) or number of cycles (first or second).

The study was conducted in compliance with the ethical standards of Resolution 466/2012 of the Brazilian National Health Council, the 1964 Helsinki declaration and its later amendments, and the recommendations set by the Strengthening the Reporting of Observational Studies in Epidemiology (STROBE) guidelines (von Elm *et al*., 2014).

The study was approved by the local Institutional Review Board, and all participating patients signed an informed consent before participating in the study.

### Study design

In this case-control prospective study, all participating patients followed the same COH using the GnRH antagonist protocol. At the moment of oocyte maturation triggering, patients were randomly allocated into two groups: group A (control group) - conventional hCG triggering; and group B (test group) GnRH agonist in association with hCG (dual triggering).

### Ovarian stimulation and triggering

Ovarian stimulation for all patients followed the same in-house protocol, always conducted by the same doctor with large experience in assisted reproductive technology (ART). A daily dose of urinary FSH (Fostimon, UCB) 300 UI was administered from day 2 of the menstruation. GnRH antagonist (Orgalutran, MSD) 0.25 mg/day was administered from the moment follicles reached 15 mm in diameter until triggering. Follicular growth was monitored with transvaginal ultrasound scans every other day until triggering criteria were met, always by the same ultrasound specialist.

Final oocyte maturation was triggered when at least two follicles measuring ≥ 17 mm were observed. Patients allocated to group A, underwent conventional triggering with two boluses of hCG (Choriomon, UCB) 5,000 UI. Patients allocated to group B underwent dual triggering with the administration of 1 ml of 5 mg leuprolide acetate (Lupron, Depot) and two boluses of hCG 5,000 UI.

All oocyte retrievals took place under transvaginal ultrasound guidance, 34-36 h after triggering, and harvested oocytes were sent to the embryology laboratory, where they were counted and classified according to the criteria established by Veeck (2002), always by the same experienced embryologist.

### Outcome variables

The outcome variables of this study were the number of total and metaphase II (MII) oocytes retrieved.

### Statistical analysis

Data collected were statistically analyzed with the assistance of *Statistica* 13.2 single user (TIBCO Statistica^®^ - Palo Alto, CA, USA). Considering the non-parametric distribution of data, the groups were compared using the Mann-Whitney test. We used the Spearman correlation test to check the correlation between the variables (total number of oocytes, MII oocytes, and patients’ age). The level of statistical significance was set at *p* ≤ 0.05.

## RESULTS

A total of 117 patients, aged between 21 and 38 years were included in the study period. A total of 50 patients were allocated to group A (conventional hCH triggering) and 67 patients to group B (dual triggering). Two patients in group A and one patient in group B had their cycles interrupted due to the absence of adequate number of follicles before triggering, and were removed from the analysis. There were no moderate or severe OHSS events in any of the patients.

**Figure 1:**
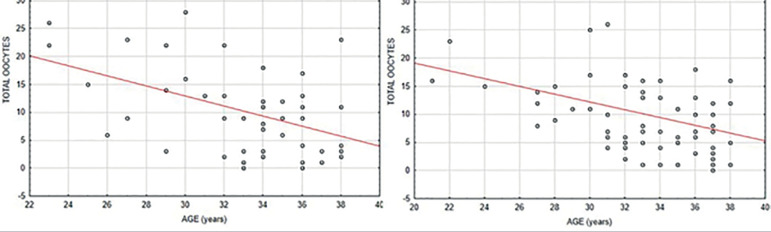
Distribution of the total number of oocytes retrieved in relation to the age of patients. A - conventional trigger (hCG); B - dual trigger (hCG + GnRH agonist)

**Figure 2:**
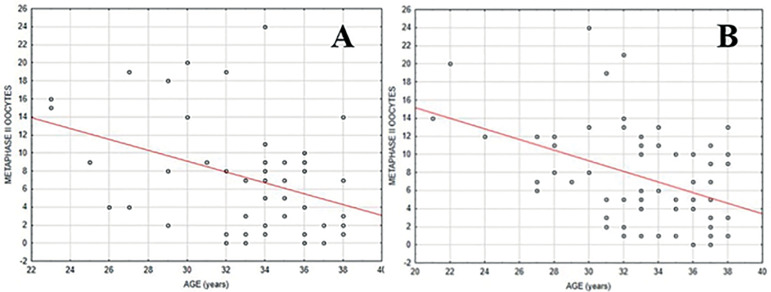
Distribution of metaphase II oocytes retrieved in relation to the age of patients. **A** - conventional trigger (hCG); **B** - dual trigger (hCG + GnRH agonist)

The two groups were homogeneous concerning the age of patients (*p* = 0.824). No statistically significant differences in the number of total (*p* = 0.604), or MII, oocytes (*p* = 0.502) retrieved were seen between the groups (Table 1). The MII/total oocyte rate was 70.9% in group A, and 74.5% in group B, with no statistically significant differences between the groups (*p* = 0.679). Only 2/48 patients (4.16%) had no oocyte retrieval in group A, when compared to 1/66 patients (1.5%) in group B (Figure 1). A total of 4/48 patients (8.3%) in group A and 2/66 (3%) in group B presented no MII oocyte retrieval (Figure 2).

**Table 1:** Mean, standard deviation (±SD), minimum and maximum values for age, total oocytes and MII oocytes obtained for group A (hCG alone) and group B (hCG+GnRH agonist).

Group	Variable	n	Mean	±	SD	Minimum	Maximum	*p* *
**A**	Age (years)	48	33.1	±	3.9	23	38	0.8248
**B**		66	33.0	±	3.8	21	38	
**A**	Total oocytes (n)	48	10.2	±	8.5	0	38	0.6041
**B**		66	10.1	±	6.9	0	38	
**A**	MII oocytes (n)	48	7.2	±	6.1	0	24	0.5022
**B**		66	7.5	±	5.4	0	24	

* Mann-Whitney test.

Age was found to be positively correlated with both the number of total and MII oocytes retrieved (*p*<0.05) (Table 2). As the patients got older the number of oocytes decreased, regardless of the triggering protocol (Figures 1 and 2).

**Table 2:** Correlation among the studied variables.

Variables	Age	Total oocytes
Total oocytes	-0.3844*	-----
Metaphase II oocytes	-0.3514*	0.9590

* Spearman correlation (*p* < 0.05).

## DISCUSSION

This study was designed to check whether any advantage in terms of increasing the total number of oocytes and MII oocytes retrieval could be obtained with the use of the dual trigger approach when compared to the conventional hCG triggering in an unselected population of Brazilian women submitted to COH regimens with GnRH antagonist. Although the results demonstrated some benefits with the use of dual trigger, there were no statistically differences between the two strategies in terms of the number of total and MII oocytes retrieved.

A possible benefit of oocyte triggering using GnRH agonists is the more physiological approach, which stimulates both mid-cycle LH and FSH surge, similarly to what occurs in an ovulating healthy woman (Prochazka *et al*., 2012). However, studies comparing conventional hCH trigger with the dual trigger approach have reported controversial results on the outcome of final oocyte maturation. Retrospective studies have shown that the number of total and mature oocytes retrieved were at least similar, and sometimes significantly higher with dual trigger (Lin *et al*., 2013; Lu *et al*., 2016; Seval *et al*., 2016, Jones *et al*., 2019). In a retrospective case-control study, Seval *et al*. (2016) demonstrated that although the mean number of total oocytes retrieved were similar, the mean number of metaphase II oocytes was significantly higher in the dual trigger group than in the hCH group. According to the authors, the higher number of MII oocytes observed with the dual trigger strategy was associated with the positive effect of FSH on pre-ovulatory follicles.

The findings of the present study seem to corroborate the perception that both strategies produce similar results in unselected patients. A non-significantly higher number of MII oocytes was retrieved with the dual trigger strategy, when compared with hCG alone. This finding is in agreement with previous prospective randomized controlled trials (RCTs), which also demonstrated that although the number of mature oocytes retrieved was higher with the dual trigger strategy than hCG alone, the difference did not reach statistical significance (Decleer *et al*., 2014; Kim *et al*., 2014; Mahajan *et al*., 2016). The same was also shown for the number of total oocytes retrieved (Schachter *et al*., 2008; Mahajan *et al*., 2016). When the data from these 4 RCTs were pooled together in two recent systematic reviews, the lack of significance between the groups in relation to the number of total and MII oocytes retrieved was confirmed (Ding *et al*., 2017; Chen *et al*., 2018).

Nonetheless, despite the lack of significance between groups, the use of dual triggering seemed to produce some benefits when compared to hCG alone. For instance, the mean MII/total oocyte rate was slightly higher with the dual trigger approach (74.5%), when compared with hCG triggering alone (70.9%). Moreover, there was also a smaller number of women with no oocytes retrieved after dual triggering, with just one patient (1.5%), than in the hCG group, with 2 patients (4.16%). The same trend was also seen for the number of women with no MII oocytes retrieved (3.0% x 8.3%). Although the findings of the present prospective study indicate that the dual trigger strategy may produce some slightly better results than hCH alone, they also confirm the lack of statistical significance between the strategies to retrieve fertilizable oocytes in unselected women undergoing GnRH antagonist IVF cycles. Figures 1 and 2 show that the total number and MII oocytes retrieved followed the same pattern regardless of the triggering approach used. Rather than the triggering protocol, age was the main factor to affect the number of oocytes retrieved, demonstrating statistical significance.

While this study indicated no improved number of retrieved oocytes obtained with the dual trigger protocol in the studied population, the approach may have an indication for patients suffering from specific conditions such as repetitive implantation failure (RIF), empty follicular syndrome, or previous poor oocyte-embryo quality (Kol & Humaidan, 2010). In a study by Pacheco *et al*. (2014), the authors suggested that dual triggering could be helpful in retrieving mature oocytes from small follicles in poor responders. Additionally, Ben-Haroush *et al*. (2019) recently suggested that dual triggering should be considered when oocyte maturation rates after conventional hCG triggering is lower than 70%. On the other hand, although dual triggering resulted in a significantly higher mean number of total oocytes and MII oocytes retrieved, it has not been recommended as the primary ovulation trigger in normal and high responders undergoing donation regimens due to the significantly higher rates of OHSS (Jones *et al*., 2019).

Some important points concerning the present study should be considered when the results are analyzed. This study was composed by all ART women referred to our service during the study period that accepted to participate in the study, regardless of age, infertility factor or number of cycles. While this sample selection approach presents the advantage of producing more generalizable findings throughout the population, it fails in accounting for more specific cases. Moreover, hCG doses used for final oocyte triggering can range from 1,500 to 2,500 IU in normo-responders, and 10,000 IU in poor-responders or in patients with hypothalamic amenorrhea (Jones *et al*., 2019). In the present study, a standard hCG dose of 10,000 IU was used in all patients regardless of oocyte response or trigger strategy, in order to reduce medication dose bias. Although, an increased risk of OHSS has been associated with hCG triggering (Delvigne & Rozenberg, 2003), no patient in the present study presented any signs of moderate or severe OHSS.

## CONCLUSION

Considering the results and the limitations of the present study, we can conclude that the dual trigger approach for final oocyte maturation is equivalent to conventional hCH triggering in terms of the number of total and MII oocytes retrieved in the general population. Further studies are necessary to ascertain dual triggering indication in selected groups of women.
